# Seasonal variation in utilization of biogenic microhabitats by littorinid snails on tropical rocky shores

**DOI:** 10.1007/s00227-012-2017-3

**Published:** 2012-08-24

**Authors:** Stephen R. Cartwright, Gray A. Williams

**Affiliations:** The Swire Institute of Marine Science and Division of Ecology & Biodiversity, The School of Biological Sciences, The University of Hong Kong, Pokfulam Road, Hong Kong SAR, China

## Abstract

Mobile species may actively seek refuge from stressful conditions in biogenic habitats on rocky shores. In Hong Kong, the upper intertidal zone is extremely stressful, especially in summer when organisms are emersed for long periods in hot desiccating conditions. As a result, many species migrate downshore between winter and summer to reduce these stressful conditions. The littorinids *Echinolittorina malaccana* and *E. vidua*, for example, are found on open rock surfaces high on the shore in winter but the majority migrate downshore in summer to the same tidal height as a common barnacle, *Tetraclita japonica*. In the laboratory, where environmental conditions could be controlled to approximate those occurring on the shore, we tested whether the downshore migration allowed littorinids to select barnacles as biogenic habitats to reduce stress and if this behaviour varied between seasons. In summer, littorinids demonstrated a strong active preference for the barnacles, which was not observed in the cool winter conditions, when animals were found on open rock surfaces even when barnacles were present. Littorinids, therefore, only actively select biogenic habitats during the summer in Hong Kong when they migrate downshore, suggesting that such habitats may play an important, temporal, role in mitigating environmental stress on tropical shores.

## Introduction

The rocky intertidal is a dynamic environmental gradient defined by variation in the duration that organisms spend submersed in seawater or emersed in air at low tide, with associated thermal and desiccation stresses (reviewed in Little et al. 2009). As a result, species are distributed along this gradient according to their ability to withstand these environmental changes (Wolcott 1973; Garrity 1984; Helmuth and Hofmann 2001). Species inhabiting thermally stressful environments utilize a variety of physiological or behavioural responses to minimize their exposure to harmful temperatures. Whilst physiological responses determine the tolerance limits of an organism (Somero 2002; Pörtner and Farrell 2008), behavioural responses such as utilizing refuges, forming aggregations or adopting postures which can minimize heat gain (Garrity 1984; Bauwens et al. 1996, Munoz et al. 2005), can reduce the physiological stress experienced by organisms. Despite these responses, periodically individuals are killed when they are in conditions which exceed their physiological tolerances (e.g. on hot summer days; Wolcott 1973; Chan et al. 2006) especially on tropical shores where species live closer to their thermal limits than their temperate counterparts (Somero 2002, 2010; Tewksbury et al. 2008).

Mobile species utilize a variety of behavioural responses to alleviate thermal stress. Mobile gastropods, for example, forage when washed by waves and then hide in cool, damp refuges or aggregate together during emersion (Garrity 1984; Williams and Morritt 1995; Chapman and Underwood 1996). Topographic features such as crevices and rockpools are typically used as refuges; however, the importance of species which act as biogenic habitats (ecosystem engineers, sensu Jones et al. 1997) is becoming increasingly acknowledged (e.g. Seed 1996; Thompson et al. 1996; Bertness et al. 1999; Castilla et al. 2004). The stalked barnacle, *Capitulum mitella,* for example, shades the rock from irradiation keeping it cool, which benefits the mobile organisms aggregating amongst them (Kawai and Tokeshi 2004). Such positive biogenic interactions can be especially important in thermally stressful conditions, such as during hot periods of the year and in tropical areas (Somero 2002; Bruno et al. 2003).

Hong Kong lies within the tropics, but experiences a strongly seasonal climate due to changes in the prevailing monsoons, resulting in a relatively cool and dry winter, and a hot and wet tropical summer (Kaehler and Williams 1996). Variation in the timing of low tides, which occur during the afternoon in the summer, means that environmental conditions are extremely stressful in summer when rock temperatures on some shores can exceed 55 °C (Williams unpublished data), and average temperatures can reach 45 °C (Williams and Morritt 1995; Cartwright 2010). As a result, intertidal assemblages show strong seasonal variation (Williams 1993; Kaehler and Williams 1996), with extensive growth of macroalgae in the winter which die back as the summer monsoon strengthens, leaving the acorn barnacle, *Tetraclita japonica japonica,* as the dominant space occupier in the midshore (~60 % cover, Chan and Williams 2004). These barnacles shade the rock surface and provide a biogenic refuge for small invertebrates (Reimer 1976; Bertness 1989). Mobile gastropods such as the limpet, *Cellana grata,* and the littorinids that live high on the shore on open rock surfaces during the winter migrate downshore in summer (Williams and Morritt 1995; Mak 1996; Harper and Williams 2001) into the barnacle dominated area, where they can utilize shade from the barnacles. Although such habitat utilization has been described (Williams and Morritt 1995, Burnaford 2004), it is often unclear whether species actively select biogenic refuges under different environmental conditions or whether differential mortality kills off animals that do not utilize these refuges (but see Jones and Boulding 1999). The present study tested whether two high shore littorinids, *Echinolittorina malaccana* and *E. vidua,* demonstrate a preference for biogenic refuges under controlled and realistic stress conditions in the laboratory. We predicted that, if refuge selection is a response to periods of increasing physiological stress, there would be greater utilization of the barnacle habitat during the hot summer months, compared to the cool winter months, when thermal stress amelioration would be less important. Furthermore, there would be a stronger selection for large barnacles as refuges that may offer more protection than small barnacles.

## Materials and methods

### Do littorinids utilize barnacles as biogenic habitats on the shore in summer and winter?

*Echinolittorina malaccana* and *E. vidua* (mean size 7.4–8.9 mm and 5.4–6.7 mm, respectively) are abundant in the high shore and splash zone of Hong Kong shores (Mak 1996). These two species forage whilst awash on the flooding and ebbing tides (Williams 1994; Stafford et al. 2007) and then seek refuges or aggregations, sealing their opercula and attaching themselves to the substrate by mucus. Whilst the distribution of the two species overlap, *E. malaccana* lives slightly higher on the shore and is more heat tolerant than *E. vidua* (LT50 of *E. malaccana* = 56.5 °C, LT50 of *E. vidua* = 54.7 °C; Marshall et al. 2011; Li 2012). To assess the abundance and distribution of littorinids in the barnacle, *Tetraclita japonica japonica* dominated area, 10-m transects horizontal to the shoreline were established at 1.75 m (where barnacles are abundant) above chart datum (C.D.) at two, randomly selected semi-exposed to exposed rocky shores in Shek O, Hong Kong (22°14′N, 114°15′E). Transects were sampled monthly in summer (June, July, August) and winter (November, December, January). At each transect, 15 randomly selected 25 × 25 cm quadrats were photographed every month (10 megapixel Canon 900TI, set at highest resolution) and the abundance of littorinids counted from the photographs (∑*n* = 2 seasons × 3 months × 2 transects × 15 quadrats = 180). In the summer, when the littorinids were most abundant in the barnacle zone, the habitats in which littorinids were found were also scored. These habitats included ‘barnacles’ (littorinid in direct contact with barnacle test); ‘bare rock surface’; ‘crack’ (depression in the rock too small for a littorinid to fit in); and ‘crevice’ (depression in the rock large enough for a littorinid to fit fully into). A Chi-square test was used to investigate whether the littorinids utilized the habitats in a proportional manner to habitat availability, with the null hypothesis that the littorinids were evenly distributed amongst the habitats.

### Habitat selection by littorinids under simulated summer and winter conditions in the laboratory

To investigate whether littorinids would actively select habitats or were simply associated with habitats dependent on relative availability, littorinids were given a choice of refuges as bare rock, small (mean ± SD, 1.5 ± 0.2 cm basal diameter, 0.5 ± 0.2 cm height) or large barnacles (3.0 ± 0.2 cm basal diameter, 2.5 ± 0.3 cm height, determined from field surveys) under controlled laboratory conditions. Small and large tests of *Tetraclita japonica japonica* were used to determine whether size of the habitat played a role in the selection decision. As refuge selection is predicted to be driven by adverse thermal conditions, the experiment was repeated in summer (July, when ambient temperatures are high) and winter (December, when temperatures are cool), to determine whether selection changes with environmental conditions or whether there is a seasonal difference in the behaviour of the littorinids.

**Fig. 1:**
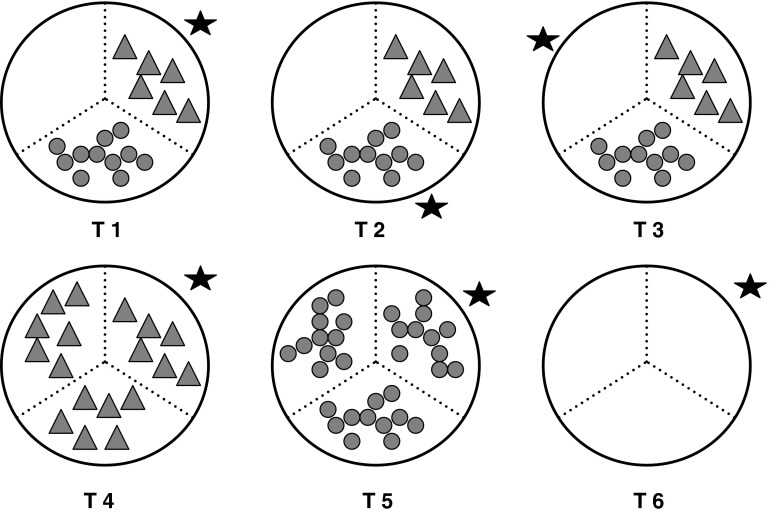
Experimental treatments (T1–T6). Each of the three arena segments is assigned a habitat: large barnacles (*triangles*), small barnacles (*circles*), and bare rock (*empty*). Treatments T1–T3 have mixed habitats, and snails are given a choice of three different habitats. Treatments T4–T6 have only one type of habitat in all three segments, and snails have no choice of habitat. The *star* denotes the segment in which animals were haphazardly placed at the beginning of the experiment

Individuals of the two littorinid species were placed separately in arenas (granite tiles with circular areas, 18 cm diameter, divided into three equal segments) with either three habitats (open rock, small barnacles and large barnacles) or each habitat in isolation (see Olabarria et al. 2002). Six treatments were established; three mixed habitat treatments (T1–T3, where littorinids were initially placed in different habitats and therefore which allowed the littorinids to choose between the original habitats they were placed in and the full range of available habitats) and three homogeneous habitats (T4–T6, where littorinids would have no choice of habitats, Fig. 1, after Olabarria et al. 2002). In the mixed treatments, each of the three types of habitats was randomly assigned to a separate segment. Open rock habitats were simply bare tile surfaces; small and large barnacle habitats were made from empty tests collected from the shore which were cleaned and fixed to the tiles with silicone glue. The arrangement of the barnacles matched a random segment taken from photoquadrats of patches of small and large barnacles on the shore. In the homogenous treatments (T4–T6), all three segments were the same habitat. In these treatments, the littorinids effectively had no choice of habitat, but these were used as controls to determine whether random dispersal of individuals would occur when there is no choice of habitat. To prevent the littorinids from escaping, Tanglefoot Treegum was applied to the edge of the circle (Tanglefoot Treegum, USA, see Davies et al. 1997). Each treatment had 15 replicates (∑*n* = 6 treatments × 2 species × 2 seasons × 15 replicates = 360).

To simulate the thermal environment experienced on the shores during summer, the arenas were randomly located in a large Perspex tidal tank (130 × 80 × 41 cm, l × w × ht) fitted with overhead lamps (6 × 200 W, Philips Halogen Plus Line Pro). When the arenas were wetted with seaspray, the arena surface temperatures were the same as seawater (~28 °C in the summer). Turning off the spray resulted in the lamps drying the rock and a gradual temperature increase, which matched that of the natural rock surface following emersion in the summer, until the rock reached 40 ± 1.5 °C (Cartwright 2010).

*Echinolittorina malaccana* (mean ± SD, 8 ± 1 mm) and *E. vidua* (mean ± SD, 7 ± 1 mm) were collected from shores where barnacles were present, transferred to the laboratory and immediately given seaspray to allow them to regain mantle water and become active. Littorinids were maintained in the laboratory for a day prior to the experiments being conducted. Experiments were repeated separately for each species and animals were randomly assigned to treatments (after Olabarria et al. 2002).

In each arena, 20 active individuals (foot extended and moving) were haphazardly placed in one of the segments, with no contact between individuals, so that any aggregations formed would be due to littorinids moving together. Animals were allowed to move freely under the seawater spray for 2 h, after which the spray was turned off to simulate the beginning of tidal emersion, and the arenas dried and surface temperatures gradually increased. Littorinids were active for the first 40 min but, as the tiles dried, they stopped moving and became inactive. After 2 h, when all littorinids were inactive, the number of individuals found in each habitat was counted.

To test whether the littorinids exhibit a preference for a particular habitat when given a choice of three different habitats (T1–T3), the proportions of littorinids that remained in, or returned to the starting segment of each treatment (Fig. 1), were analysed by one-way ANOVA (six treatments, fixed factor). To control for random refuge selection, any preference shown in treatments (T1–T3) should correspond to similar proportions in (T4–T6). Therefore, if there is a higher proportion of littorinids in T1 in the starting segment by the end of the experiment compared to T2 and T3, then T4 should also have higher proportions in the starting segment than T5 and T6 (refer to Olabarria et al. 2002 for more details). Preferences, if any, were determined for each season separately (one-way ANOVA), but to test whether preference for habitat was more evident in either season, a two-way ANOVA (∑*n* = 3 treatments × 2 seasons × 15 replicates = 90) was used to test between seasons (2 levels = summer and winter, fixed and orthogonal) and treatments (3 levels = T1–T3, fixed and orthogonal). Only treatments T1–T3 were used, as once preference within a season was established, the control treatments (T4–T6) became irrelevant.

As littorinids are known to form aggregations to reduce environmental stress (Garrity 1984, Chapman and Underwood 1996, Stafford 2002), to determine whether a habitat affected the degree of aggregation, the number of individuals in aggregations (individuals in contact with two or more other littorinids, Stafford 2002) was scored within different habitats. To disassociate the influence of having a choice of habitats, only the homogeneous treatments were scored (T4–T6). Data were analysed separately for each species, in each season using a two-way ANOVA with treatments (fixed factor, 3 levels = T4–T6) and seasons (fixed factor). Proportional data were arcsin transformed and analyses were run using WinGmav 5 (EICC, The University of Sydney). Homogeneity of variances was checked using Cochran’s test (Underwood 1997) and significant differences for fixed factor effects further analysed by SNK tests.

## Results

### On-shore species abundance and distribution amongst habitats during summer and winter

**Fig. 2:**
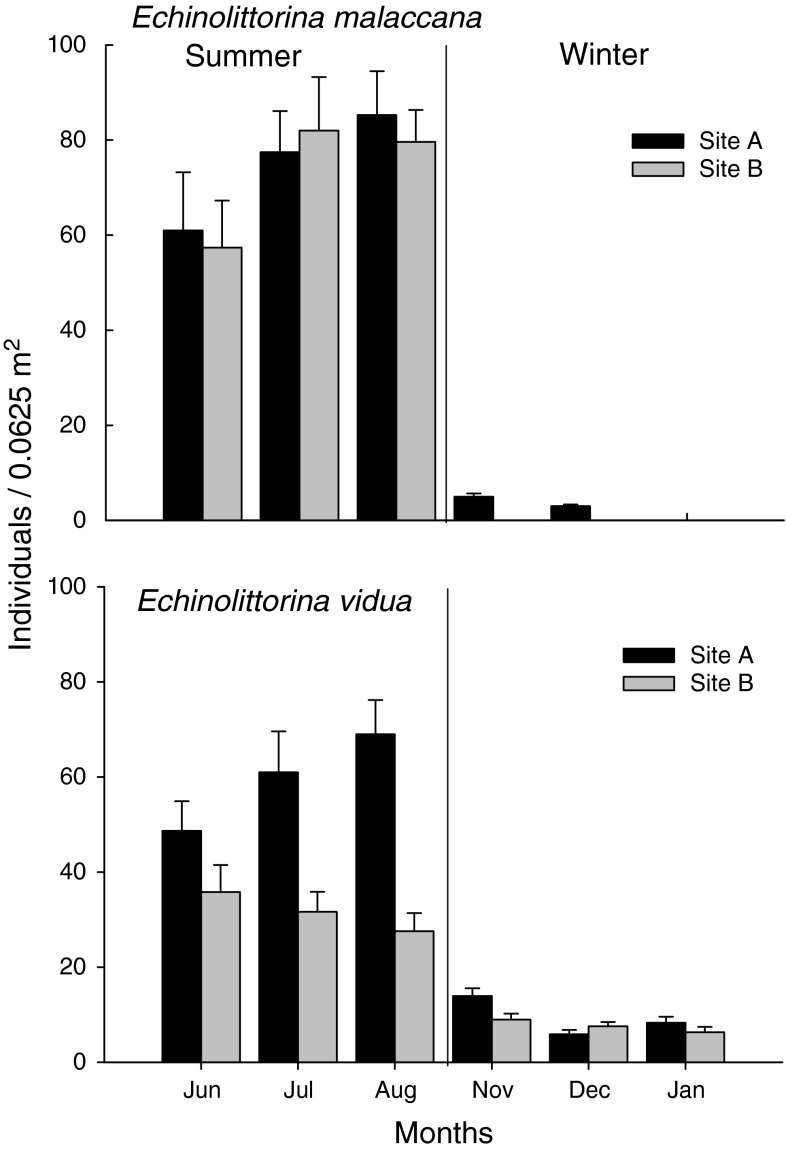
Mean abundance (+SE) of *Echinolittorina malaccana* and *E. vidua* individuals in the barnacle habitats (25 × 25 cm quadrats, *n* = 15) at two sites in Shek O during 3 months in summer and 3 months in winter

**Fig. 3:**
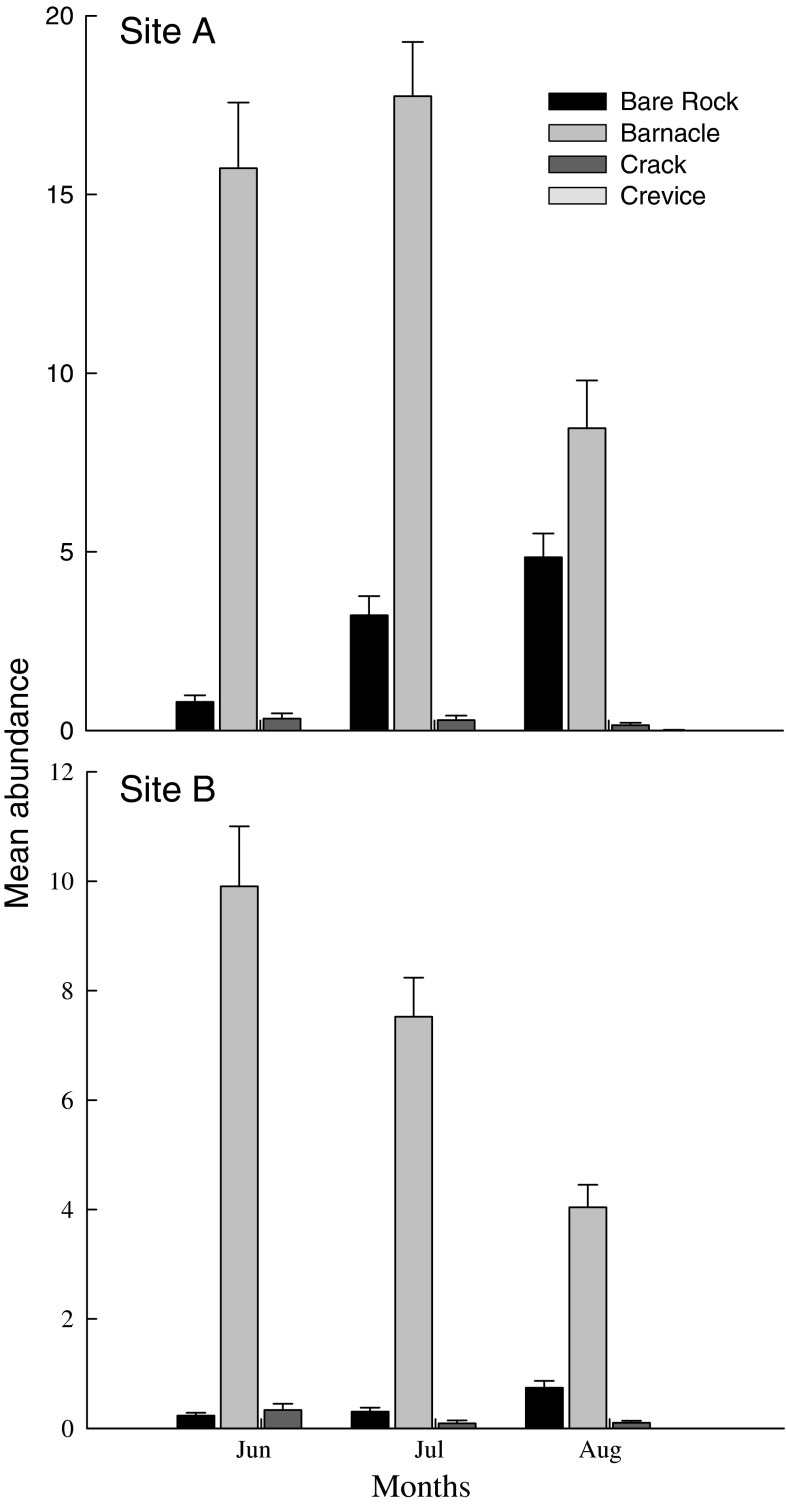
Mean (+SE) abundance of littorinids found in each habitat in 25 × 25 cm quadrats (*n* = 180) for the months of June, July and August at site A and B

In the winter, littorinids were found ~0.5 m above the barnacle zone, whereas in summer they were much more abundant lower on the shore within the barnacle zone at both sites, especially for *Echinolittorina malaccana* (Fig. 2). During summer >90 % of the individuals were associated with barnacle tests as opposed to other available habitats such as bare rock (Fig. 3, ${{\chi}_{2,3}}$ = 9,381.6, *P* < 0.05), despite the fact that the mean barnacle cover on these shores was only ~35–40 % (Cartwright 2010).

### Habitat selection by littorinids under simulated stressful conditions in the laboratory during summer and winter

**Fig. 4:**
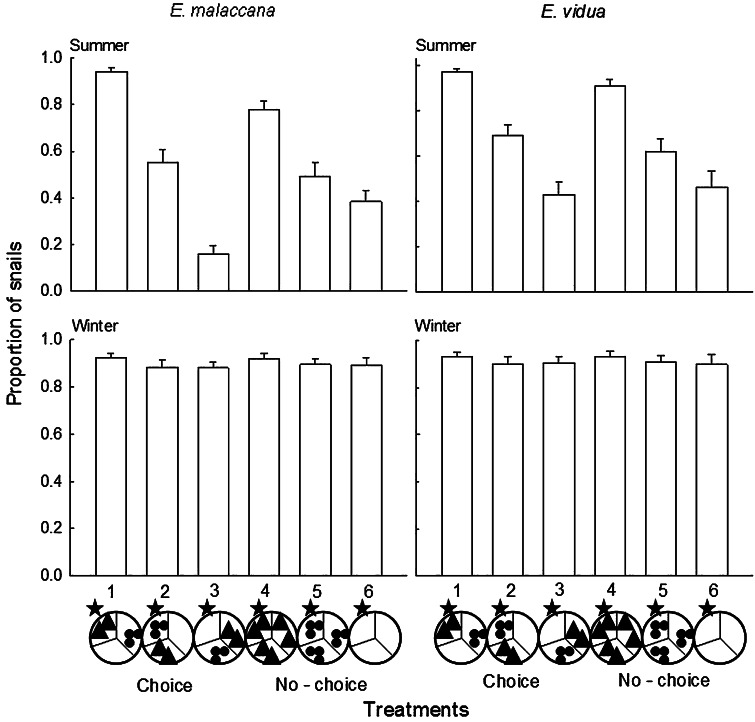
Mean proportion (+SE) of *Echinolittorina malaccana* and *E. vidua* remaining in their original refuges (position denoted by a *star*) in different treatments (T1–T6) at the end of each experiment (*n* = 15) during summer and winter. For explanations of the treatments, see Fig. 1

There was no significant difference between treatments in winter as littorinids tended to remain in the habitats they were originally placed in, suggesting no habitat preference (Table 1, Fig. 4). Distribution of both *Echinolittorina malaccana* and *E. vidua,* however, varied between treatments during summer (Table 1). In summer, both species showed an overall trend to avoid open rock surfaces and to associate with large and small barnacles (Fig. 4), often moving into the empty barnacle tests. Mixed habitats with snails starting in the large barnacles and homogeneous, no choice, large barnacle treatments had a significantly higher proportion of both species remaining in, or returning to, their original habitat as compared to all the other treatments (Fig. 4). In the other treatments, littorinids left their starting habitats (small barnacles or bare rock) and moved to use large barnacles as habitats when a choice was available or dispersed randomly when there was no choice (Fig. 4).

**Table 1:**
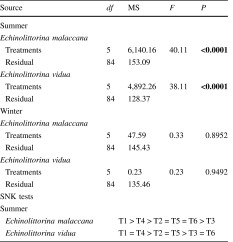
One-way analyses of variance to investigate variation in the distribution of *Echinolittorina malaccana* and *E. vidua* between different habitats in summer and winter

Littorinids were given a choice of habitats (mixed;* T1* starting in large barnacles;* T2* starting in small barnacles;* T3* starting in bare rock, refer to Fig. 1) or no choice (homogeneous;* T4* large barnacles only;* T5* small barnacles only;* T6* bare rock only, refer to Fig. 1); (∑*n* = 6 treatments; fixed factor ×15 replicates = 90). Proportional data were arcsin transformed. Variances were homogenous (Cochran’s test: *P* < 0.05). Significant interactions (*P* < 0.05; in bold) were further analysed using Student–Newman–Keuls (SNK) post hoc tests

**Fig. 5:**
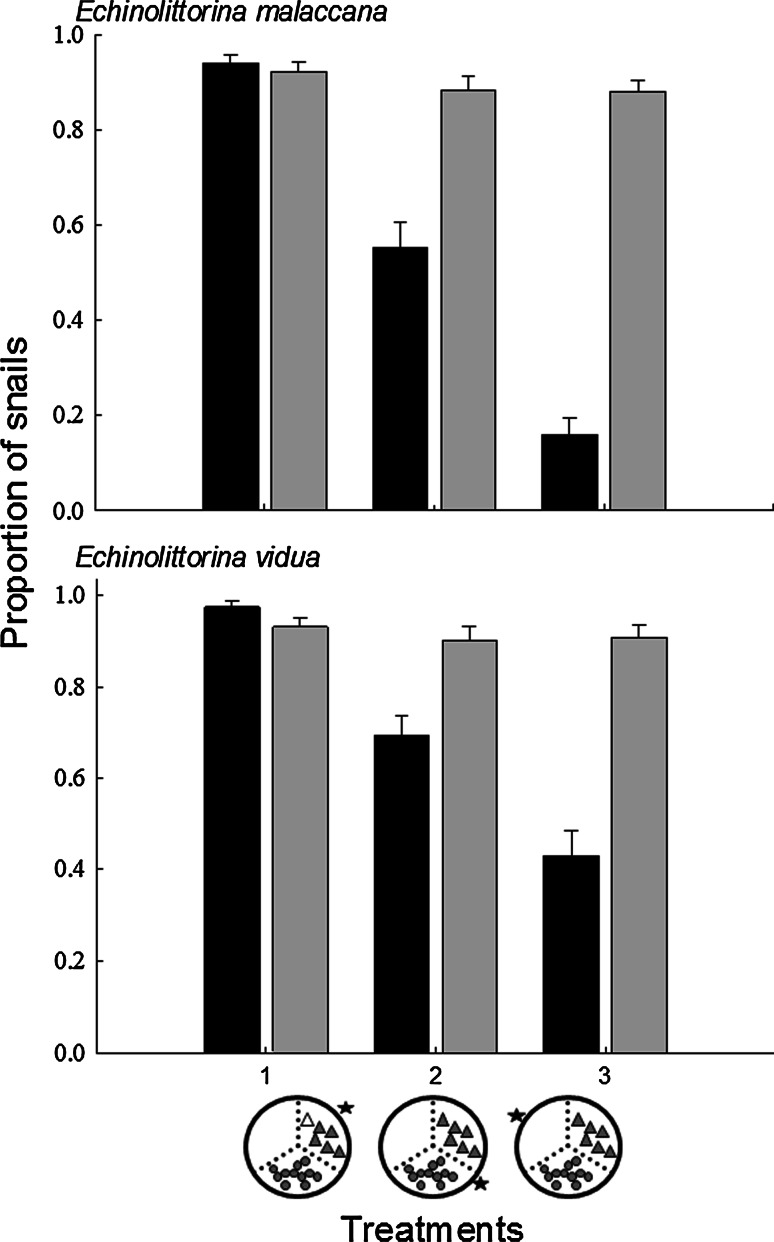
Mean proportion (+SE) of *Echinolittorina malaccana* and *E. vidua* remaining in their original refuges (position denoted by a *star*) in different choice treatments (T1–T3) at the end of each experiment (*n* = 15), between summer (*black bars*) and winter (*grey bars*). (For explanations of the treatments, see Fig. 1)

When analysing choice amongst different habitats, there was a significant interaction between seasons and treatments for both *Echinolittorina malaccana* and *E. vidua* (Table 2, Fig. 5). In general, in the winter, there was no difference in the proportions of littorinids moving out of the habitats between littorinids originally placed in small barnacles or open rock habitats. Littorinids placed in large barnacle habitats, however, did not move from their original habitats, or if they did, they later returned to these habitats (Fig. 5). In contrast, during summer, the littorinids exhibited significant differences in proportions in each habitat, showing a preference amongst refuges in the order large barnacle > small barnacle > bare rock (Table 2).

**Table 2:** Two-way analyses of variance to investigate variation in habitat preference for *Echinolittorina malaccana* and *E. vidua*, between summer (S) and winter (W) when given a choice of habitats (T1–T3) (∑n = 2 seasons; fixed factor × 3 treatments; fixed factor ×15 replicates = 90)

Source	*df*	MS	*F*	*P*
*Echinolittorina malaccana*				
Season	1	14,835.43	105.34	**<0.0001**
Treatment	2	7,158.08	50.82	**<0.0001**
Season × treatment	2	5,407.09	38.39	**<0.0001**
Residual	84	140.84		
*Echinolittorina vidua*				
Season	1	5,609.72	80.88	**<0.0001**
Treatment	2	3,987.27	36.17	**<0.0001**
Season × treatment	2	3,379.21	30.65	**<0.0001**
Residual	84	110.25		
SNK tests				
*Echinolittorina malaccana*	T1 W = S		W T1 = T2 = T3	
	T2 W > S		S T1 > T2 > T3	
	T3 W > S			
*Echinolittorina vidua*	T1 W = S		W T1 = T2 = T3	
	T2 W > S		S T1 > T2 > T3	
	T3 W > S			

Proportional data were arcsin transformed. Variances were homogenous (Cochran’s test: *P* < 0.05). Significant interactions (*P* < 0.05; in bold) were further analysed using Student–Newman–Keuls (SNK) post hoc tests

### Aggregation behaviour

**Fig. 6:**
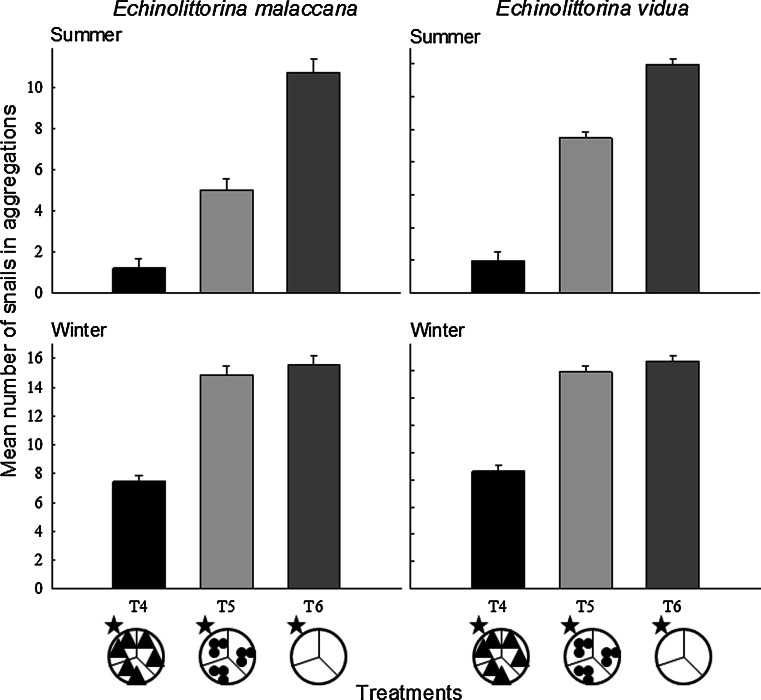
Mean number (+SE) of *Echinolittorina malaccana* and *E. vidua* individuals in aggregations within different habitats (T4–T6, position denoted by a *star*; *n* = 15), during summer and winter

In winter, the haphazardly placed littorinids tended to form aggregations, and few solitary individuals were found. In summer, some aggregations were initially formed, but as the temperature increased over time, these aggregations tended to dissociate, as individuals dispersed and finally became inactive in different habitats (S.R. Cartwright pers. obs.). In winter, however, littorinids formed permanent aggregations, moving short distances to come into contact and remain with other individuals regardless of which habitat they were in, resulting in a tendency for littorinids to remain in the same segment that they were initially placed in. In both summer and winter, there was a trend for more individuals of both species to aggregate in the bare rock treatment, followed by the small barnacles and least in the large barnacle treatment (Table 3). In winter, fewer individuals (<50 %) of either species aggregated in the large barnacle treatment as compared to small barnacle and bare rock treatments which were similar (Table 3), with over 50 % of the littorinids being found in aggregations (Fig. 6). In summer, the number of individuals aggregating was lowest in the large barnacle treatment, followed by the small barnacle treatment, and then the largest number of individuals aggregated together in the bare rock treatment (Table 3, Fig. 6). *Echinolittorina vidua* tended to aggregate more than *E. malaccana* in the small barnacle treatment, although in the large barnacle treatment, both species showed low aggregation tendencies (Table 3, Fig. 6).

**Table 3:** Two-way analyses of variance to investigate variation in the numbers of *Echinolittorina malaccana* and *E. vidua* in aggregations when given no choice of habitats (homogeneous; T4: larger barnacles; T5: small barnacles; T6: bare rock) in summer and winter (∑*n* = two seasons, fixed factor and three treatments; fixed factor ×15 replicates = 90)

Source	*df*	MS	*F*	*P*
*Echinolittorina malaccana*				
Season	1	1102.50	237.22	**<0.0001**
Treatment	2	599.21	128.93	<**0.0001**
Season × treatment	2	49.9	10.74	**<0.001**
Residual	84	4.65		
Total	89			
*Echinolittorina vidua*				
Season	1	624.10	218.19	**<0.0001**
Treatment	2	811.811	283.82	**<0.0001**
Season × treatment	2	36.23	12.67	**<0.0001**
Residual	84	2.86		
Total	89			
SNK tests				
*Echinolittorina malaccana*				
Season (treatment):	Treatment (season):			
T4: Summer < Winter	Summer: T4 < T5 < T6			
T5: Summer < Winter	Winter: T4 < T5 = T6			
T6: Summer < Winter				
*Echinolittorina vidua*				
Season (treatment):	Treatment (season)			
T4: Summer < Winter	Summer: T4 < T5 < T6			
T5: Summer < Winter	Winter: T4 < T5 = T6			
T6: Summer < Winter				

Variances were homogenous (Cochran’s test: *P* < 0.05). Significant factors (*P* < 0.05; in bold) were further analysed using Student–Newman–Keuls (SNK) post hoc tests

## Discussion

Both *Echinolittorina malaccana* and *E. vidua* were strongly associated with barnacle tests during summer when temperature stress is high. At this time of year, temperature stress can be lethal to many other species on the shores (Morton 1995; Chan et al. 2006; Williams unpublished data). In summer, when given an equal choice of small barnacles, large barnacles and open rock, in controlled laboratory conditions, littorinids demonstrated a preference for the large barnacles, stopping in locations such as the interstices between two barnacle tests, on the sides of the barnacle test, and in some cases, crawling inside the barnacle tests. These locations provided shade to the organisms and thus reduced the amount of radiating light (and heat) received (Denny and Harley 2006). Such benefits would be absent from the open rock surfaces, and even small barnacles which did not offer much shielding from direct insolation. Conversely, in winter, littorinids showed no selection, even under conditions of thermal stress which would normally be experienced in the summer, and were more likely to stop in open areas or form aggregations. This seasonal pattern in habitat choice may be driven by the highly contrasting seasonal climate which Hong Kong experiences (Kaehler and Williams 1996). More importantly, summer rock temperatures may exceed 55° C (Williams unpublished data), and the timing of the lowest tides occurs during the early afternoons in summer, as opposed to early morning in the winter, leading to a seasonal ‘hotspot’ when extreme stresses occur in the intertidal zone (Helmuth et al. 2002). The aggregating behaviour observed may be a response to water conservation in the dry conditions during winter as opposed to avoiding insolation.

Migration downshore by littorinids (and other gastropods) prior to the summer months is a well-described pattern on the seasonal, tropical shores of Hong Kong (Williams and Morritt 1995; Mak 1996; Harper and Williams 2001), which reduces emersion times, and therefore physical stress the species experience. Migrating downshore brings littorinids to the same shore height that barnacles inhabit. At this level, the barnacles increase surface heterogeneity (Kostylev et al. 2005) and can provide a potential refuge for mobile species to select. During tidal emersion, many gastropods isolate themselves from the environment by retracting their foot into their shells to minimize their water loss (Britton and McMahon 1990; Ng 2007). Where these animals spend their emersion period inactive is, however, also critical in determining their risk to thermal and desiccation stress (Williams and Morritt 1995), as selecting habitats which mitigate environmental stresses will further decrease this risk (Garrity 1984). Refuge selection is, therefore, an important behavioural strategy to add to the variety of responses individuals can use to withstand thermal stress (Somero 2002, 2010) which may help organisms to stay within the optimum range of their thermal windows (Pörtner and Farrell 2008).

Littorinids are thought to form aggregations to reduce temperature and evaporative water loss (Chapman and Underwood 1996; Stafford 2002). In summer, however, when physiological stress is high, individuals initially formed aggregations in the laboratory, but later moved to barnacle refuges. This suggests that mechanisms that result in the downshore migration of these littorinid species (Mak 1996; Harper and Williams 2001) may also drive these animals to seek barnacle refuges, at times when aggregation with conspecifics alone may not be sufficient to cope with the environmental conditions.

Intertidal ecosystem engineers, such as algae (Bertness et al. 1999), mussels (Seed 1996), and barnacles in temperate regions (Thompson et al. 1996), have been suggested to increase species diversity and abundance through reduction of environmental stresses (Jones and Boulding 1999; Harley and O’Reily 2011). These barnacles also provide shade that can reduce the amount of direct solar insolation experienced by individuals which is a key component of an individual’s heat energy budget (Kawai and Tokeshi 2004; Denny and Harley 2006). There is, however, a lack of empirical data to establish whether association of mobile species with biogenic habitats is an active response (but see Jones and Boulding 1999), and often, the mechanisms by which the organisms benefit are more anecdotal than explicitly tested (Bulleri 2009). This study demonstrated that species actively select biogenic refuges during times of the year that conditions were environmentally stressful, but not during environmentally benign times of the year. Animals may, therefore, only use biogenic habitats at certain times when conditions are stressful, whilst for the rest of the year, they are able to exploit other areas without the need to seek refuge. Such temporally important roles of biogenic habitats and seasonal variation in species behaviour patterns, therefore, are important in determining the integrated success and fitness of a species. This is especially true in regions which experience strong seasonal variation in environmental conditions and where the loss of the biogenic habitats may have cascading effects on assemblage structure (Crain and Bertness 2005).

## References

[CR1] Bauwens D, Hertz PE, Castilla AM (1996). Thermoregulation in a Lacertid lizard: the relative contributions of distinct behavioural mechanisms. Ecology.

[CR2] Bertness MD (1989). Intraspecific competition and facilitation in a northern acorn barnacle population. Ecology.

[CR3] Bertness MD, Leonard GH, Levine JM, Schmidt PR, Ingraham AO (1999). Testing the relative contribution of positive and negative interactions in rocky intertidal communities. Ecology.

[CR4] Britton JC, McMahon RF (1990) The relationship between vertical distribution, evaporative water loss rate, behaviour, and some morphometric parameters in four species of rocky intertidal gastropods from Hong Kong. In: Morton B (ed) Proceedings of the 2nd international marine biological workshop: the marine flora and fauna of Hong Kong and Southern China, Hong Kong. Hong Kong University Press, Hong Kong, pp. 1153–1171

[CR5] Bruno JF, Stachowicz JJ, Bertness MD (2003). Inclusion of facilitation into ecological theory. Trends Ecol Evol.

[CR6] Bulleri F (2009). Facilitation research in marine systems: state of the art, emerging patterns and insights for future developments. J Ecol.

[CR7] Burnaford JL (2004). Habitat modification and refuge from sublethal stress drive a marine plant-herbivore association. Ecology.

[CR8] Cartwright SR (2010) Facilitation of intertidal species against environmental stress by the barnacle *Tetraclita japonica japonica* on Hong Kong’s tropical rocky shores. PhD Thesis, The University of Hong Kong, Hong Kong

[CR9] Castilla JC, Lagos NA, Cerda M (2004). Marine ecosystem engineering by the alien ascidian *Pyura praeputialis* on a mid-intertidal rocky shore. Mar Ecol Prog Ser.

[CR10] Chan BKK, Williams GA (2004). Population dynamics of the acorn barnacles, *Tetraclita squamosa* and *Tetraclita japonica* (Cirripedia: Balanomorpha), in Hong Kong. Mar Biol.

[CR11] Chan KK, Morritt D, De Pirro M, Leung KMY, Williams GA (2006). Summer mortality: effects on the distribution and abundance of the acorn barnacle *Tetraclita japonica* on tropical shores. Mar Ecol Prog Ser.

[CR12] Chapman MG, Underwood AJ (1996). Influences of tidal conditions, temperature and desiccation on patterns of aggregation of the high-shore periwinkle, *Littorina unifasciata*, in New South Wales, Australia. J Exp Mar Biol Ecol.

[CR100] Crain CM, Bertness MD (2005) Community impacts of a tussock sedge: is ecosystem engineering important in benign habitats? Ecology 86:2695–2704

[CR13] Davies MS, Knowles AJ, Edmonston P, Hutchinson N (1997). The use of a commercial insect-trapping compound to maintain grazer densities on rocky shores. Trans Nat Hist Soc North.

[CR14] Denny MW, Harley CDG (2006). Hot limpets: predicting body temperature in a conductance-mediated thermal system. J Exp Biol.

[CR15] Garrity SD (1984). Some adaptations of gastropods to physical stress on tropical rocky shores. Ecology.

[CR16] Harley CDG, O’Reily JL (2011). Non-linear density-dependent effects of an intertidal ecosystem engineer. Oecologia.

[CR17] Harper KD, Williams GA (2001). Variation in abundance and distribution of the chiton *Acanthopleura japonica* and associated molluscs on a seasonal, tropical, rocky shore. J Zool.

[CR18] Helmuth B, Hofmann GE (2001). Microhabitats, thermal heterogeneity, and patterns of physiological stress in the rocky intertidal zone. Biol Bull.

[CR19] Helmuth B, Harley CDG, Halpin PM, O’Donnell M, Hofmann GE, Blanchette CA (2002). Climate change and latitudinal patterns of intertidal thermal stress. Science.

[CR20] Jones KMM, Boulding EG (1999). State-dependent habitat selection by an intertidal snail: the costs of selecting a physically stressful microhabitat. J Exp Mar Biol Ecol.

[CR21] Jones CG, Lawton JH, Shachak M (1997). Positive and negative effects of organisms as physical ecosystem engineers. Ecology.

[CR22] Kaehler S, Williams GA (1996). Distribution of algae on tropical rocky shores: spatial and temporal patterns of non-coralline encrusting algae in Hong Kong. Mar Biol.

[CR23] Kawai T, Tokeshi M (2004). Variable modes of facilitation in the upper intertidal: goose barnacles and mussels. Mar Ecol Progr Ser.

[CR24] Kostylev VE, Erlandsson J, Mak YM, Williams GA (2005). The relative importance of habitat complexity and surface area assessing biodiversity: fractal application on rocky shores. Ecol Comp.

[CR25] Li HTK (2012) Thermal tolerance of *Echinolittorina* species in Hong Kong: implications for their vertical distributions. MPhil Thesis, The University of Hong Kong, Hong Kong

[CR26] Little C, Williams GA, Trowbridge CD (2009). The biology of rocky shores.

[CR27] Mak YM (1996) The ecology of high-zoned littorinids, *Nodilittorina trochoides*, *N. radiata* and *N. vidua* on rocky shores in Hong Kong, PhD Thesis, The University of Hong Kong, Hong Kong

[CR28] Marshall DJ, Dong YW, McQuaid CD, Williams GA (2011). Thermal adaptation in the intertidal snail *Echinolittorina malaccana* contradicts current theory by revealing the crucial roles of resting metabolism. J Exp Biol.

[CR29] Morton B (1995). The population dynamics and reproductive cycle of *Septifer virgatus* (Bivalvia, Mytilidae) on an exposed rocky shore in Hong Kong. J Zool.

[CR30] Munoz JLP, Finke GR, Camus PA, Bozinovic F (2005). Thermoregulatory behaviour, heat gain and thermal tolerance in the periwinkle *Echinolittorina peruviana* in central Chile. Comp Bio Physiol.

[CR31] Ng JSS (2007) Resource partitioning and coexistence of molluscan grazers on Hong Kong rocky shores. PhD Thesis, The University of Hong Kong, Hong Kong

[CR32] Olabarria C, Underwood AJ, Chapman MG (2002). Appropriate experimental design to evaluate preferences for microhabitat: an example of preferences by species of microgastopods. Oecologia.

[CR33] Pörtner HO, Farrell AP (2008). Physiology and climate change. Science.

[CR34] Reimer AA (1976). Succession of invertebrates in vacant tests of *Tetraclita stalactifera panamensis*. Mar Biol.

[CR35] Seed R (1996). Patterns of biodiversity in the macro-invertebrate fauna associated with mussel patches on rocky shores. J Mar Biol Assoc UK.

[CR36] Somero GN (2002). Thermal physiology and vertical zonation of intertidal animals: optima, limits, and costs of living. Integr Comp Biol.

[CR37] Somero GN (2010). The physiology of climate change: how potentials for acclimatization and genetic adaptation will determine ‘winners’ and ‘losers’. J Exp Biol.

[CR38] Stafford R (2002) The role of environmental stress and physical and biological interactions on the ecology of high shore littorinids in a temperate and a tropical region. PhD Thesis, University of Sunderland, Sunderland, UK

[CR39] Stafford R, Davies MS, Williams GA (2007). Computer simulations of high shore littorinids predict small-scale spatial and temporal distribution patterns on rocky shores. Mar Ecol Prog Ser.

[CR101] Tewksbury JJ, Huey RB, Deutsch CA (2008) Putting the heat on tropical animals. Science 320:1296 10.1126/science.1159328 18535231

[CR40] Thompson RC, Wilson BJ, Tobin ML, Hill AS, Hawkins SJ (1996). Biologically generated habitat provision and diversity of rocky shore organisms at a hierarchy of spatial scales. J Exp Mar Biol Ecol.

[CR41] Underwood AJ (1997). Experiments in ecology: their logical design and interpretation using analysis of variance.

[CR42] Williams GA (1993). Seasonal variation in algal species richness and abundance in the presence of molluscan herbivores on a tropical rocky shore. J Exp Biol Ecol.

[CR43] Williams GA (1994). The relationship between shade and molluscan grazing in structuring communities on a moderately-exposed tropical rocky shore. J Exp Mar Biol Ecol.

[CR44] Williams GA, Morritt D (1995). Habitat partitioning and thermal tolerance in a tropical limpet, *Cellana grata*. Mar Ecol Progr Ser.

[CR45] Wolcott TG (1973). Physiological ecology and intertidal zonation in limpets (*Acmaea*): a critical look at “limiting factors”. Biol Bull.

